# Surface Ligand-Dependent Kinetics of α‑Lactalbumin Interaction with Gold Nanoparticles Investigated by SPR

**DOI:** 10.1021/acsomega.6c01893

**Published:** 2026-09-07

**Authors:** Carlos Antônio Ferraz, Isabela Araujo Marques, Thalles Maranesi Pereira, Ygor Ribeiro Guimarães, Cínthia das Dores Aguiar, Hauster Maximiler Campos de Paula, Luciano Sindra Virtuoso, Nelson Henrique Teixeira Lemes, Ana Clarissa dos Santos Pires, Luis Henrique Mendes da Silva, Yara Luiza Coelho Zampier

**Affiliations:** † Grupo de Pesquisa em Química de Coloides, Instituto de Química, 74347 Universidade Federal de Alfenas, Alfenas 37130-000, Brasil; ‡ Advanced Thermokinetics of Molecular Systems (ATOMS) Group, Departamento de Química, Universidade Federal de Viçosa, Viçosa 36570-000, Brasil; § Laboratório de Termodinâmica Molecular Aplicada (THERMA), Departamento de Tecnologia de Alimentos, Universidade Federal de Viçosa, Viçosa 36570-000, Brasil

## Abstract

Understanding the kinetic and energetic factors governing protein adsorption onto nanomaterial surfaces is essential for elucidating nanobiointerfacial processes. In this study, the interaction between α-lactalbumin (αLa) and gold nanoparticles (AuNPs) functionalized with different surface ligands (citrate, 3-mercaptopropionic acid (MPA), and polyethylene glycol (PEG 6000)) was investigated by surface plasmon resonance (SPR). Real-time monitoring enabled determination of association and dissociation rate constants, residence times, and activation parameters over a controlled temperature range (285.2–301.2 K). Adsorption proceeded under a rapid kinetic regime for all systems, with both association (10^7^–10^8^ M^–1^ s^–1^) and dissociation (0.5 s^–1^) occurring on short time scales. Despite this overall similarity, the magnitude and thermal dependence of kinetic parameters were strongly influenced by ligand chemistry, with association rates and residence times following the order PEG > MPA ≈ citrate. Activation analyses indicated that formation of the transition state possibly involves contributions from interfacial desolvation and counterion release. For citrate-coated nanoparticles, temperature-dependent parameters were obtained, suggesting the involvement of conformational changes on αLa upon binding. These findings provide mechanistic insights into how surface functionalization governs the kinetic and energetic pathways of protein adsorption at gold nanobiointerfaces.

## Graphical abstract

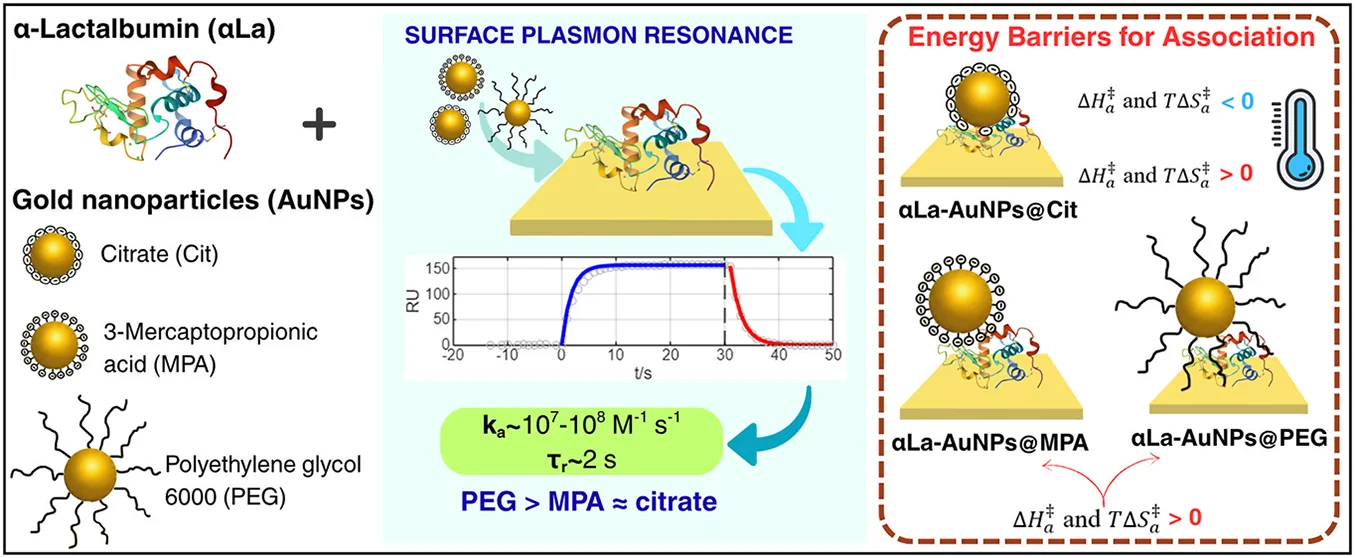

## 1 Introduction

The rapid expansion of nanotechnology has intensified scientific interest in interactions occurring between nanomaterials and biological systems, particularly those established between metallic nanoparticles (NPs) and proteins. Such interactions modulate interfacial physicochemical properties and colloidal behavior in biological environments,^1–3^ thereby governing performance in applications ranging from biosensing^4,5^ to nanomedicine.^6,7^

When metallic nanoparticles are introduced into biological fluids, including blood or interstitial media, their surfaces are rapidly coated by biomolecules, predominantly proteins, resulting in the loss of their original synthetic identity and the emergence of a new biological identity.^8,9^ This adsorbed layer, known as the protein corona, is hierarchically organized and composed of protein populations exhibiting distinct binding affinities and residence times. From kinetic and thermodynamic perspectives, this interfacial architecture can be subdivided into a strongly bound fraction (hard corona) and a dynamically exchangeable fraction (soft corona), reflecting the coexistence of interactions spanning multiple energetic and temporal regimes.^10–12^

Among the various nanomaterials investigated, gold nanoparticles (AuNPs) occupy a prominent position owing to their unique optical properties, high biocompatibility, synthetic versatility, and extensive surface functionalization capability.^13–15^ From a biophysicochemical standpoint, AuNPs exhibit pronounced affinity toward proteins, arising from the combined contributions of electrostatic forces, van der Waals interactions, hydrophobic effects, and, in specific systems, coordination between thiol groups of cysteine residues and the gold surface.^16,17^ This interaction is highly dependent on surface coating: Cationic CTAB-AuNPs exhibit very high affinities toward proteins such as bovine serum albumin (BSA) and fibrinogen (FIB) (*K_a_* ∼ 10^8^–10^9^ M^–1^), forming dense coronas and inducing structural perturbations.^18^ In contrast, small anionic ligands (e.g., citrate, DHLA) promote electrostatic adsorption and conformational rearrangements, whereas further functionalization with PEG or zwitterionic groups markedly suppresses corona formation, highlighting ligand charge, chain length, and grafting density as key regulators of nano–bio interfacial thermodynamics and protein stability.^19^

Surface functionalization dictates AuNP–protein interactions, making structurally well-defined biomacromolecules essential for mechanistic adsorption studies. Within this framework, milk proteins are widely employed as model systems and as functional materials in food and biomedical applications.^20,21^ α-lactalbumin (αLa) constitutes a particularly informative protein for investigating nanoparticle adsorption mechanisms. This small globular whey protein (∼14.2 kDa) contains four disulfide bonds and a calcium-binding loop essential for maintaining its native conformation.^22,23^ Perturbations in structural stability, whether induced by environmental factors or interfacial adsorption, can expose hydrophobic domains and reactive residues, thereby modulating binding affinity toward nanostructured surfaces. For example, interaction with 30 nm AuNPs induces partial unfolding, conferring the protein higher interfacial activity and greater penetration capacity into anionic and zwitterionic phospholipid monolayers.^24^ Moreover, studies show that biosynthesized AuNPs may act as promising therapeutic agents against amyloidosis; at a concentration of 2 μg/mL, a 46% inhibition of αLa amorphous aggregation was observed, effectively preventing the α-helix to cross-β conformational transition typical of fibril formation.^25^ Such structural responsiveness renders αLa a sensitive probe for evaluating how nanoparticle surface chemistry influences adsorption kinetics.

In this context, surface plasmon resonance (SPR) emerges as a particularly powerful analytical platform for investigating nanobio interfacial interactions. SPR enables real-time, label-free monitoring of adsorption processes while providing access to kinetic and thermodynamic parameters.^26,27^ Despite its extensive application in biomolecular interaction studies, systematic investigations focusing on the influence of nanoparticle surface functionalization on αLa adsorption kinetics remain limited. Accordingly, the present work aims to fill this gap by providing a kinetic analysis of αLa interactions with AuNPs with different coating (citrate, 3-mercaptopropionic acid, and poly­(ethylene glycol)), elucidating the physicochemical mechanisms governing complex formation.

## 2 Materials and Methods

### 2.1 Materials

Chloroauric acid (HAuCl_4_·*x*H_2_O, 99%), sodium citrate, polyethylene glycol (PEG) with average number molecular weight (*M_n_*) of 6000 g·mol^–1^, 3-mercaptopropionic acid (MPA, 99%), and αLa were purchased from Sigma-Aldrich and used as received without further purification. Ultrapure water (Milli-Q, 18.2 MΩ·cm) was used in all solution preparations. All reagents were of analytical grade. CM5 sensor chips and amine coupling reagents, N-ethyl-*N*′-(3-(dimethylamino)­propyl)­carbodiimide (EDC), *N*-hydroxysuccinimide (NHS), and ethanolamine hydrochloride, were obtained from GE Healthcare (USA). All chemicals employed in SPR experiments were analytical grade and used without additional purification.

### 2.2 Synthesis and Characterization of Gold Nanoparticles

Gold nanoparticles (AuNPs) were synthesized in aqueous medium using the classical Turkevich method, based on the reduction of Au^3+^ ions by sodium citrate, yielding spherical nanoparticles with an average diameter of approximately 15 nm. Citrate-coated AuNPs (AuNPs@Cit) were used directly after synthesis. Surface functionalization of AuNPs@Cit was carried out via ligand exchange through incubation with PEG 6000 or MPA, promoting partial substitution of citrate molecules adsorbed on the nanoparticle surface.

The optical characterization of the synthesized AuNPs with different coatings (Cit, MPA and PEG 6000) was carried out by ultraviolet–visible (UV–vis) absorption spectroscopy using a Biochrom Libra S22 spectrophotometer. The hydrodynamic diameter and size distribution of the nanoparticles were determined by dynamic light scattering (DLS) using a Malvern Zetasizer Nano ZS equipped with a 633 nm He–Ne laser operating at 4 mW. Surface charge measurements were performed by zeta potential analysis using the same instrument, in order to assess the colloidal stability of the nanoparticle dispersions.

The morphology and surface distribution of AuNPs were examined by scanning electron microscopy (SEM) using a TESCAN MIRA 4 LMU field-emission scanning electron microscope (FE-SEM) equipped with a Schottky field emitter (maximum accelerating voltage of 30 kV and spatial resolution of 1.2 nm). Prior to analysis, a drop of the AuNPs@Cit suspension was deposited onto a clean silicon wafer (or conductive carbon tape mounted on an aluminum stub) and allowed to dry at room temperature. Owing to the intrinsic conductivity of gold nanoparticles, no additional conductive coating was required.

### 2.3 Surface Plasmon Resonance Analysis

SPR measurements were performed using a Biacore X100 instrument (GE Healthcare, Pittsburgh, PA, USA). CM5 sensor chips composed of carboxymethylated dextran matrices covalently bound to a gold film were employed. Each chip contained two independent flow channels: sample (with protein immobilization) and reference no protein immobilization. Detector response was monitored as a function of time, generating sensorgrams expressed in resonance units (RU), where 1 RU corresponds to an angular shift of 0.0001° in the SPR signal. Analytical response was obtained by subtracting the reference channel signal from that of the sample channel, minimizing bulk refractive index effects and nonspecific interactions.

#### 2.3.1 Immobilization of α-Lactalbumin on the Sensor Surface

αLa on CM5 chips was performed via covalent amine coupling using the standard carbodiimide activation protocol. Initially, the sensor surface was activated by injection of a freshly prepared EDC (0.4 M)/NHS (0.1 M) mixture (1:1 v/v) at a flow rate of 10 μL·min^–1^ for 7 min. Subsequently, α-lactalbumin solution (30 μg mL^–1^) prepared in 10 mM sodium acetate buffer (pH 4.0) was injected at 10 μL min^–1^ for 7 min, yielding immobilization densities of approximately 4009 RU. This immobilization level was selected to minimize mass transport limitations during interaction experiments. Remaining activated carboxyl groups were blocked by injection of ethanolamine hydrochloride (1 M, pH 8.5) for 7 min at the same flow rate.

#### 2.3.2 Interaction Analysis between AuNPs and Immobilized α-Lactalbumin

For interaction experiments, AuNP dispersions prepared previously were diluted in running buffer (pH 7.4) immediately prior to analysis. Three nanoparticle systems were investigated: AuNPs@Cit, AuNPs@MPA, AuNPs@PEG. Nanoparticle dispersions were injected over the αLa functionalized surface for 20 s at a flow rate of 7 μL·min^–1^. After each interaction cycle, surface regeneration was performed by injecting running buffer twice for 30 s at 30 μL·min^–1^, allowing removal of adsorbed nanoparticles and reuse of the immobilized protein surface in subsequent cycles.

#### 2.3.3 Data processing

The fitting procedure was divided into two distinct steps for each individual sensorgram, allowing the association and dissociation phases to be analyzed independently

1. Association Phase: Each curve was fitted individually in its local association window (*t*_on_ ≤ *t* ≤ τ) using a nonlinear least-squares algorithm (lsqcurvefit in MATLAB) to solve the pseudo-first-order eq (eq 1)­
1 $$\mathrm{RU}\left(t\right)={\mathrm{RU}}_{\max}\left(t_{\infty }\right)\left[1-e^{-k_{\mathrm{obs}}\left(t\right)}\right]$$
  where RU­(*t*) and RU_max_ (*t*_∞_) reflect, respectively, the number of complexes formed at a given *t* and at *t* = ∞. This yielded an independent parameter, the observed rate constant (*k*_obs_), for each analyte concentration ([AuNPs]). The association rate constant (*k*_a_) was then accurately extracted from the slope of the linear regression of *k*_obs_ versus [AuNPs] (*k*_obs_= *k*_a_[AuNPs] + *k*_d_).
2. Dissociation Phase: Each curve was fitted individually in its local dissociation window (*t* ≤ τ) using liner method. To eliminate the influence of the initial injection lag or buffer-switching artifacts, the dissociation phase was linearized using a logarithmic transformation (eq 2).

2 $$\ln\left(\mathrm{RU}\left(t\right)\right)=-k_{d}t+\ln\left(\mathrm{RU}\left(t_{m}\right)\right)$$

where RU­(*t*_m_) is the SPR signal at the beginning of the dissociation step, at *t_m_*, and *k*_*d*_ is the dissociation rate constant. In linear regression via the least-squares method (polyfit in MATLAB), the slope (angular coefficient) is calculated based on the global trend of the data points. If a slight delay or deviation occurs at the beginning, its impact on the slope of a long straight line is drastically smaller than it would be on an exponential curve. The final *k*_d_ reported is the average of these independently fitted stable curves.

In this procedure, points located close to the transition zone or in regions where the signal approached the baseline noise (*R* < 0.5 RU) were excluded.

The statistical procedures used for data treatment, model fitting, parameter estimation, and error propagation are described in detail in the Supporting Information.

## 3 Results and Discussion

### 3.1 Synthesis and Characterization of AuNPs

Preparation of AuNPs has been carried out in aqueous medium using the classical Turkevich method of reduction of chloroauric acid by trisodium citrate, in which Cit is acting as both reducing agent and stabilizer. Change in color from colorless to wine red was observed, indicating the formation of AuNPs@Cit. After the incubation with MPA or PEG 6000, no change in color occurred. The size of AuNPs were determined from the maximum absorption wavelength of the UV–vis spectra according to the method proposed by Haiss et al.^28^ Figure 1(a) show that all samples exhibit a maximum absorption at approximately 526 nm, which is characteristic of small, spherical gold nanoparticles with an average diameter of ∼15 nm. Morphological characterization was further performed by SEM (Figure S1).

**Figure 1:**
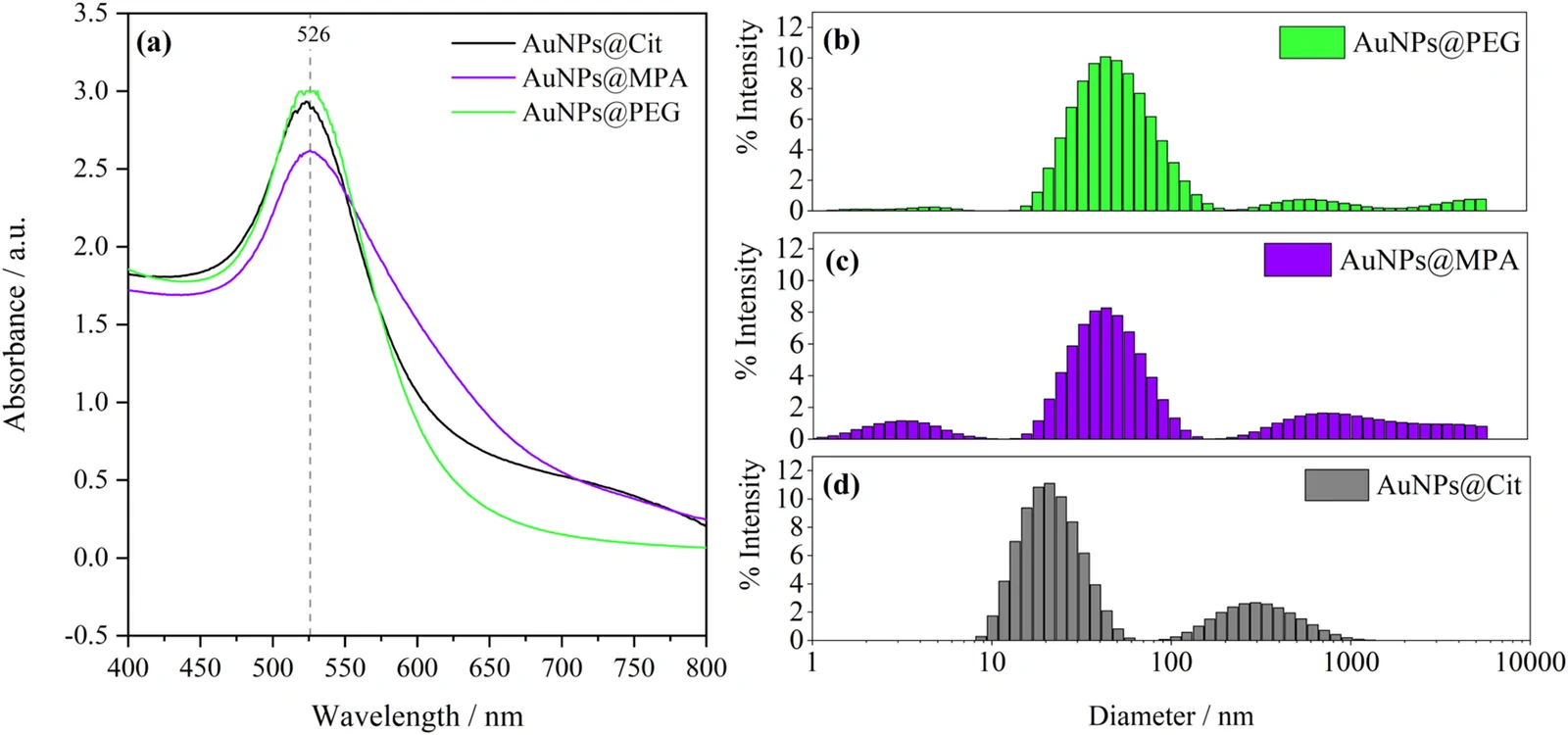
(a) UV–vis absorption spectra of AuNPs suspension (5.1 × 10^–9^ mol L^–1^) functionalized with Cit, MPA, and PEG, showing an LSPR band at ∼526 nm. (b–d) Size distributions obtained by DLS for AuNPs@PEG, AuNPs@MPA, and AuNPs@Cit, respectively, presented as intensity-weighted distributions.

DLS measurements provided complementary characterization of the AuNPs through determination of the hydrodynamic diameter (*d_h_*), polydispersity index (PDI), and zeta potential (ζ), with the corresponding size distributions shown in Figure 1b. AuNPs@Cit exhibited the smallest *d_h_* (25.6 ± 1.2 nm) and the most negative ζ value (−68.4 ± 2.3 mV), indicating strong electrostatic stabilization provided by adsorbed citrate ions. The relatively low PDI (0.369 ± 0.018) suggests a comparatively homogeneous particle population. Replacement of citrate by MPA increased *d_h_* to 41.5 ± 4.9 nm and reduced the magnitude of ζ to −36.7 ± 4.3 mV. This behavior is consistent with the formation of an MPA layer on the Au surface through Au–S interactions, resulting in partial screening of the surface charge. The higher PDI (0.578 ± 0.085) indicates greater heterogeneity in particle size and/or surface coverage. PEGylation further increased *d_h_* to 54.5 ± 0.8 nm, reflecting the formation of a hydrated polymer corona around the nanoparticles, resulting in a relatively broad hydrodynamic size distribution (PDI = 0.452 ± 0.034). The ζ value decreased substantially to −9.1 ± 2.7 mV, indicating that electrostatic repulsion becomes less important after PEG adsorption.

### 3.2 Kinetics of αLa Interaction with Surface-Functionalized AuNPs by SPR

The kinetic characterization of nanoparticle–protein interactions constitutes a central step toward elucidating the molecular events governing nanobiointerface formation, particularly with respect to recognition, adsorption, and interfacial rearrangement processes. In this context, SPR was employed as a label-free optical technique enabling real-time monitoring and determination of association (*k_a_*), and dissociation (*k_d_*) rate constants in complex biomolecular systems. The interaction kinetics between immobilized αLa, yielding a SPR response of 4009 RU corresponding to an estimated surface density of 400.9 ng cm^–2^, and AuNPs bearing distinct surface functionalizations (citrate, MPA, and PEG 6000) were systematically investigated under this framework. Representative sensorgrams acquired at 298.2 K, shown in Figure 2, constitute the experimental basis for kinetic parameter extraction and mechanistic interpretation.

**Figure 2:**
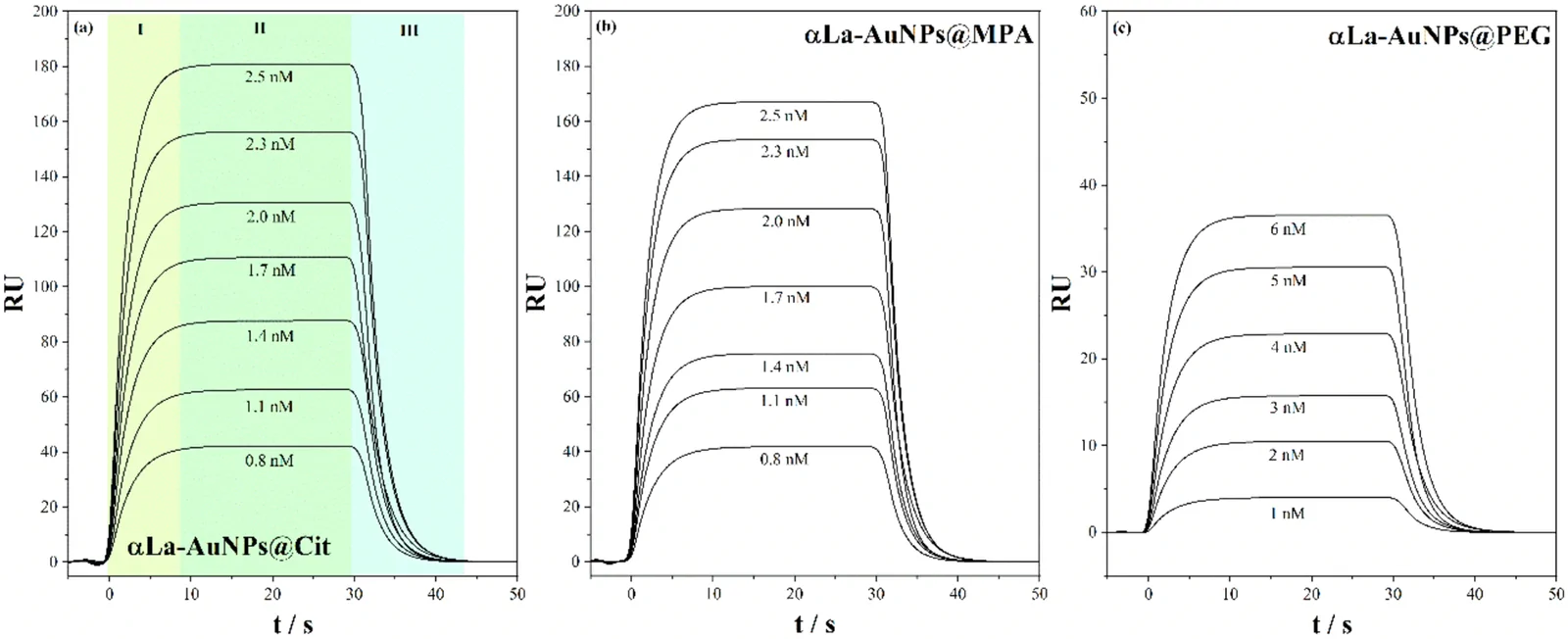
SPR sensorgrams for the interaction between immobilized αLa and surface-functionalized AuNPs at 298.2 K. (a) αLa-AuNPs@Cit; (b) αLa-AuNPs@MPA; (c) αLa-AuNPs@PEG. Increasing nanoparticle concentrations are indicated in each panel. The sensorgrams display the three characteristic kinetic stages: association (I), surface saturation (II), and dissociation (III) following buffer injection.

The temporal response profiles consisted of three well-defined stages: (I) Rapid initial association, (II) progressive surface saturation as nanoparticle concentration increases, and (III) dissociation following replacement of analyte by running buffer. This behavior was consistently observed for the αLa-AuNPs@Cit, αLa-AuNPs@MPA, and αLa-AuNPs@PEG systems, with analogous profiles recorded across the investigated temperature range, as shown in Figures S2–S4 of the Supporting Information.

Sensorgram analysis was conducted assuming that αLa immobilized on the dextran matrix interacts reversibly with AuNPs injected in solution under negligible mass-transport limitation (MTL). The MTL model accounts for the hydrodynamic phenomena within the SPR flow cell, where the mass transport coefficient (*k*_*t*_) describes the rate at which the analyte travels from the bulk solution to the sensor chip surface.^29^ If surface binding (*k*_*a*_) is significantly faster than transport (*k*_*t*_), the reaction operates under a diffusion-limited regime; conversely, if transport is instantaneous compared to binding, the classic 1:1 model is appropriate.

To test the regime of our system, a local sensitivity analysis via finite differences was performed on both *k*_*a*_ and *k*_*t*_ using the 1.7 nM sensorgram. A tiny 0.1% perturbation above the optimal *k*_*a*_ value led to a drastic shift in the simulated kinetic profile, yielding a derivative norm of 8580. This high magnitude shows that the optimization algorithm is highly sensitive to *k*_*a*_, allowing its estimation with high statistical confidence.

On the other hand, an identical 0.1% increase in *k*_*t*_ exerted a negligible effect on the curve, producing a difference norm of only 5.9 (approximately 1450 times lower than the impact observed for *k*_*a*_). This numerical evidence demonstrates that the sensorgrams are highly insensitive to mass transport. It confirms that the system is strictly under kinetic control, with mass transport playing a marginal, secondary role. Therefore, based on these statistical and hydrodynamic insights, the classic Langmuir model was preferred to avoid parameter overparameterization (overfitting). Figure 3 presents the experimental and mathematically fitted SPR sensorgrams for the interaction between immobilized αLa and surface-functionalized AuNPs@MPA at 285.2 K. (Figure 3).

**Figure 3:**
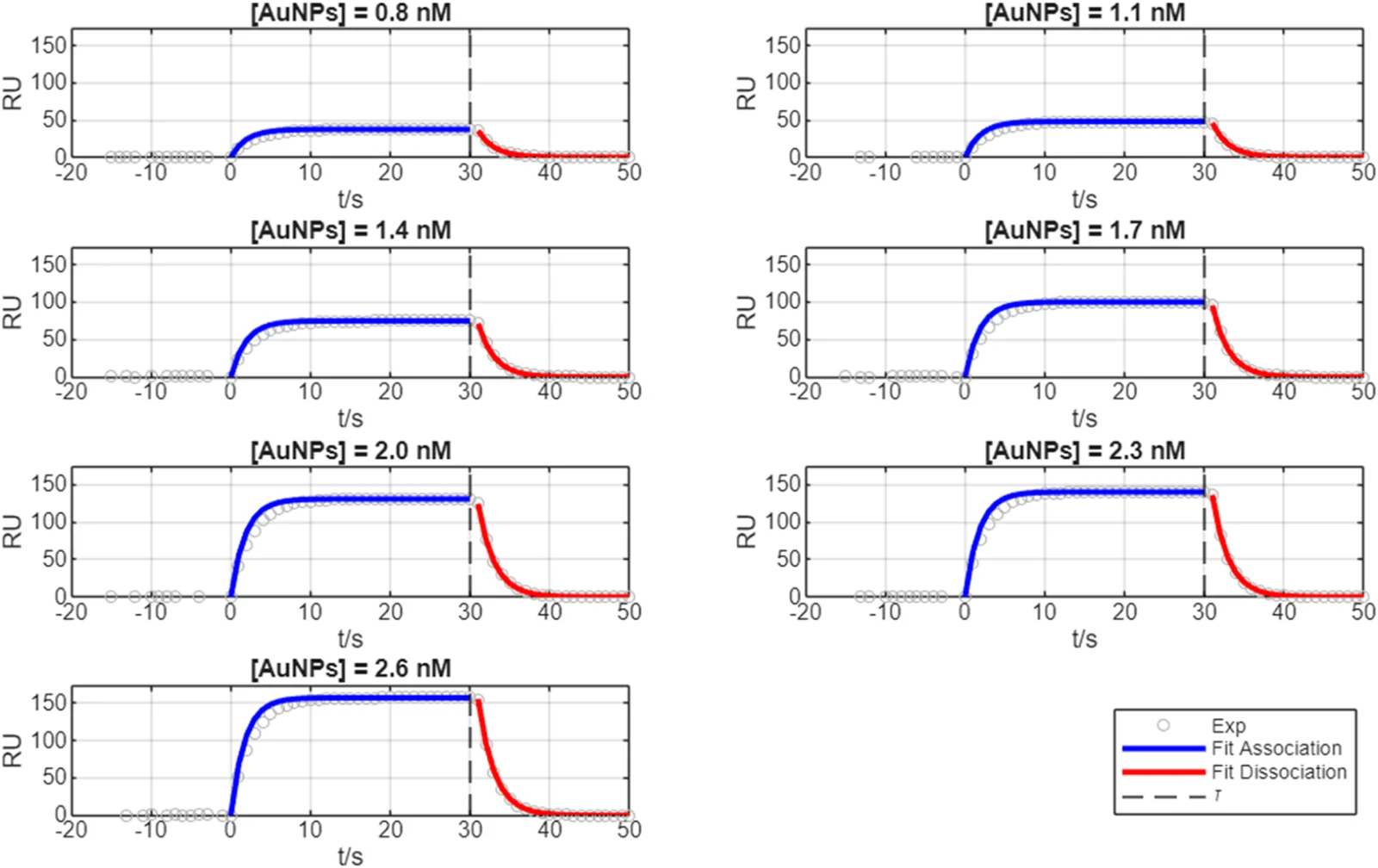
Experimental and mathematically fitted SPR sensorgrams for the interaction between immobilized αLa and surface-functionalized AuNPs@MPA at 285.2 K. Individual panels display the kinetic profiles across an increasing concentration gradient of nanoparticles (0.8 to 2.6 nM). Gray circles represents the raw experimental data RU vs *t*/*s*. The solid blue line denotes the global-local nonlinear regression fit for the association phase (0 < *t* ≤ τ). The solid red line represents the nonlinear regression fit for the dissociation phase (*t* > τ). The vertical dashed line explicitly indicates the injection matrix switching time (τ = 30s) marking the transition from surface binding to buffer-induced dissociation.

However, the most critical and definitive test to evaluate the adequacy of a kinetic model is the analysis of its residual plots, which display the point-by-point difference between the experimental data and the mathematically fitted curves (RU_exp_–RU_calc_). For a physically accurate model, the residuals must exhibit a random, scattered distribution centered around zero (white noise) across both the association and dissociation phases. This behavior indicates that the model successfully captured the true underlying physics of the phenomenon.

The residual plots along with the experimental and mathematically fitted SPR sensorgrams for the interaction between immobilized αLa and surface-functionalized AuNPs@MPA at 285.2 K are shown in Figure 4. The normal, scattered distribution of data points tightly distributed around the zero-baseline (white noise character) across all panels validates the statistical adequacy of the decoupled Langmuir model in capturing the interfacial binding kinetics. Consequently, the 1:1 kinetic model applied herein serves as an effective macroscopic approximation, and the extracted parameters, *k*_*a*_ (Table 1) and *k*_*d*_, should be interpreted as apparent kinetic constants that globally describe the interaction behavior, rather than absolute microscopic mechanistic quantities.

**Figure 4:**
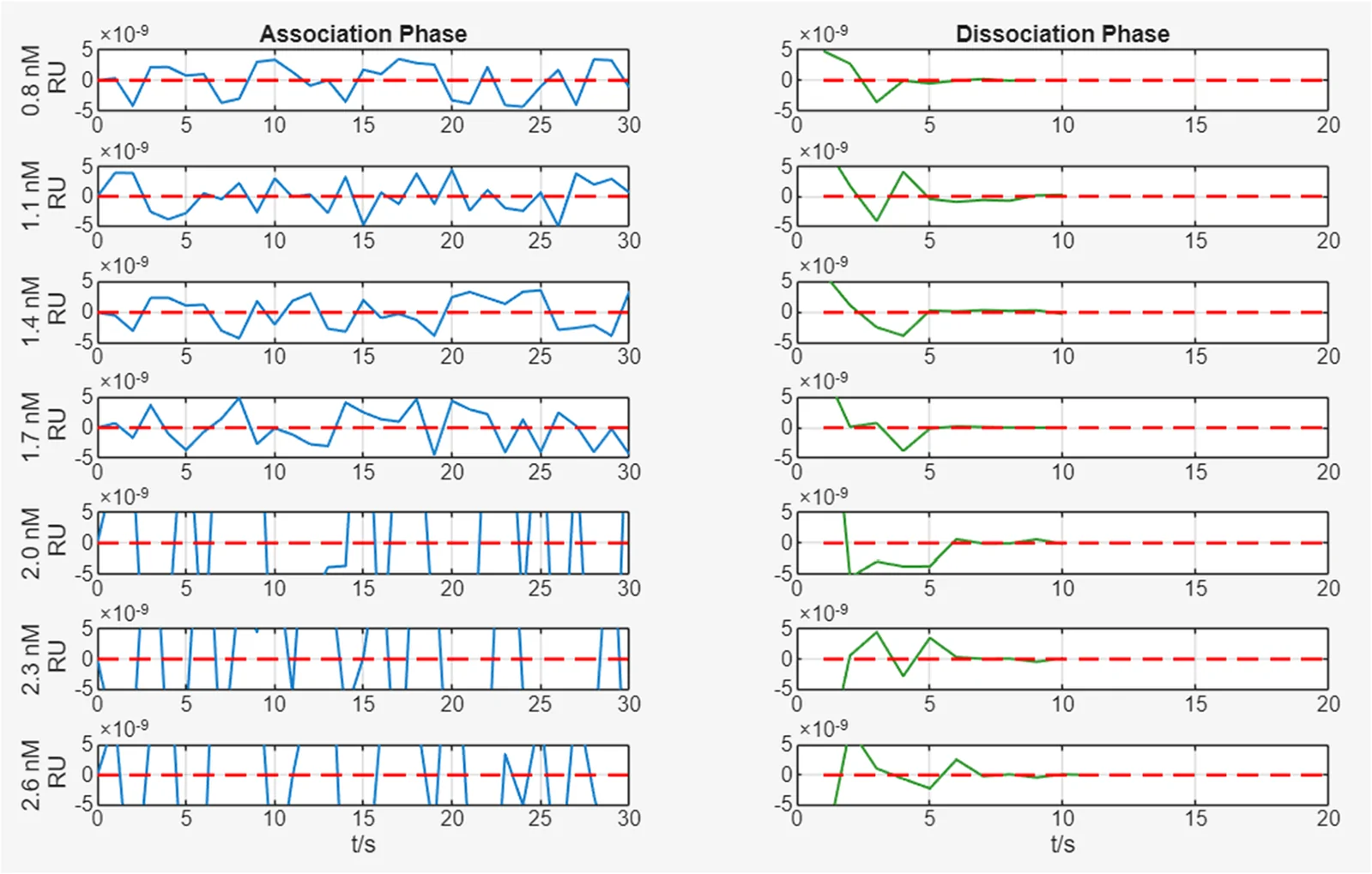
Residual plots (ΔRU = RU_exp_–RU_calc_) derived from the decoupled 1:1 kinetic interaction model between immobilized αLa and surface-functionalized AuNPs@MPA at 285.2 K. Left panels display the point-by-point residuals for the local nonlinear regression of the association phase (0 ≤ *t* ≤ 30 s). Right panels display the residuals for the log–linearized regression of the dissociation phase (*t* > 30s).

**Table 1:** Temperature Dependence of *k*_*a*_ for the Interaction between αLa and Surface-Functionalized Gold Nanoparticles (AuNPs@Cit, AuNPs@MPA, and AuNPs@PEG), Determined by SPR

	*k*_*a*_/10^7^ M^–1^s^–1^		
*T*/K	αLa–AuNPs@Cit	αLa–AuNPs@MPA	αLa–AuNPs@PEG
285.2	4.07 ± 0.06	2.62 ± 0.12	9.37 ± 0.32
289.2	5.20 ± 0.08	3.45 ± 0.09	12.1 ± 0.4
293.2	6.46 ± 0.09	4.40 ± 0.09	15.1 ± 0.6
297.2	7.26 ± 0.29	6.06 ± 0.06	18.3 ± 0.3
298.2	7.36 ± 0.09	7.02 ± 0.08	19.1 ± 0.3
301.2	7.17 ± 0.08	8.38 ± 0.07	21.3 ± 0.3

Regardless of surface functionalization, αLa-AuNP complex formation exhibited *k*_a_ on the order of 10^7^–10^8^ M^–1^ s^–1^, characterizing a fast kinetic regime. Despite the overall rapid kinetics, a clear dependence on nanoparticle surface chemistry was observed, with association rates following the predominant trend PEG > Citrate ≈ MPA throughout most of the investigated temperature range.

Interpretation of these differences must consider the electrostatic landscape under experimental conditions (pH 7.4). At this pH, terminal carboxylate groups from citrate and MPA are fully deprotonated, imparting negative surface charge to the nanoparticles. Simultaneously, αLa exhibits net negative charge given its isoelectric point (pI ≈ 4.2).^30^ This electrostatic repulsion imposes an energetic penalty during the initial approach stage, effectively increasing the activation barrier for complex formation and contributing to the relatively lower *k*_a_ values observed for anionic coatings. In contrast, PEGylated nanoparticles lack formal surface charge, avoiding electrostatic penalties, and probably promoting transient intermolecular contacts through hydrogen bonding and hydrophobic interactions.

Previous studies have reported that the passivation efficiency of PEG-coated nanoparticles depends on both PEG molecular weight and surface organization. In some systems, increasing PEG molecular weight has been associated with reduced resistance to protein adsorption, whereas shorter PEG chains have been shown to provide more effective surface passivation.^31,32^ Under these conditions, small proteins may partially penetrate the PEG corona and access exposed regions of the gold surface, thereby promoting adsorption. Consistent with this interpretation, Perera et al. reported that increasing PEG molecular weight reduced the passivation efficiency of PEG-coated AuNPs toward small biomolecules, as well as large proteins containing.^33^ Given the relatively small molecular mass of α-lactalbumin (∼14 kDa), it is plausible that the PEG layer did not completely prevent access to the AuNP surface. Instead, the flexible PEG corona may have allowed αLa to approach the nanoparticle interface and establish direct interactions with the gold surface, contributing to the higher adsorption levels observed experimentally.

Dissociation rate constants were found to be around 0.5 s^–1^ for all systems, indicating moderate complex stability and relatively dynamic interfacial exchange. To provide a more intuitive descriptor of complex persistence, the inverse dissociation constant was expressed as residence time (τ_r_ = 1/*k*_d_) and listed in Table 2.

**Table 2:** Temperature Dependence of the Residence Time (τ_r_) of the αLa-AuNPs Complexes Formed with Differently Functionalized Surfaces, Obtained from Kinetic Dissociation Parameters Derived by SPR, within the 285.2-301.2 K Temperature Interval^a^

	τ_*r*_/s		
*T*/K	αLa–AuNPs@Cit	αLa–AuNPs@MPA	αLa–AuNPs@PEG
285.2	2.15	2.11	2.30
289.2	2.22	2.06	2.25
293.2	2.23	2.00	2.17
297.2	2.16	1.93	2.12
298.2	2.12	1.91	2.11
301.2	1.96	1.87	2.06

a The associated errors (∼10^–11^s) refer exclusively to the mathematical standard deviation of the nonlinear regression fit.

Residence times were confined to a narrow temporal window (∼1.87–2.30 s), evidencing moderately stable yet dynamically reversible adsorption. Within this interval, a systematic dependence on surface functionalization was again observed. PEG-coated nanoparticles exhibited the highest τ_*r*_ values across the entire temperature range, indicating slightly more persistent complexes. Conversely, the lowest residence times were recorded for MPA-functionalized nanoparticles, suggesting comparatively weaker or less persistent adsorption. Citrate-coated AuNPs displayed intermediate behavior between these two extremes. A gradual decrease in τ_*r*_ with increasing temperature was observed for all surface chemistries. This trend reflects thermally induced destabilization of the adsorbed complexes, whereby increased molecular motion facilitates desorption and accelerates dissociation kinetics.

To date, no specific kinetic studies describing αLa interactions with nanomaterials have been reported. Nevertheless, analogous protein-AuNP systems provide useful benchmarks. For example, the interaction between human α-thrombin and 2 nm AuNPs functionalized with 4-mercaptobenzoic acid exhibited association constants on the order of 10^6^ M^–1^ s^–1^ by SPR and 10^7^ M^–1^ s^–1^ by stopped-flow spectroscopy.^34^ Notably, the residence time of the α-thrombin-AuNPs complex (∼16 s) was approximately seven times higher than that observed here for αLa-AuNPs@PEG. Although adsorption in both cases occurs with high *k*_a_ values, the differences in τ_*r*_ likely reflect intrinsic disparities in nanoparticle size, ligand packing density, and protein structural properties.

### 3.3 Activation Thermodynamics of Complex Formation

Elucidating the influence of nanoparticle surface chemistry on protein-NPs complexation kinetics requires quantitative characterization of the energetic barriers governing the process. These barriers extend beyond diffusional activation and initial intermolecular recognition, encompassing subsequent interfacial events such as desolvation, ligand shell rearrangement, and conformational accommodation of the protein at the nanoparticle surface. Complementarily, dissociation processes are dictated by the depth of the free energy well related to the stable complex formed and are therefore equally sensitive to ligand chemical identity and thermodynamic conditions.

Activation parameters associated with the association of free species (*x* = *a*) or dissociation of the stable complex were determined from temperature-dependent kinetic data. Initially, activation Gibbs free energy (Δ*G*_*x*_^‡^) was calculated over the entire investigated temperature range (eq 3). Arrhenius plots (ln *k_a_* vs 1/*T*) were subsequently constructed to obtain activation energies (*E*_*x*_^‡^) through the slope of the corresponding curves (eq 4), from which activation enthalpy (Δ*H*_*x*_^‡^) and entropy (Δ*S*_*x*_^‡^) contributions were calculated using eqs 5–6.

3 $${\Delta G}_{x}^{‡}=-\mathrm{RT}\ln\left(\frac{k_{x}h}{k_{B}T}\right)$$

4 $$E_{x}^{‡}=-R\frac{\partial \ln k_{x}}{\partial \left(1/T\right)}$$

5 $${\Delta H}_{x}^{‡}=E_{x}^{‡}-\mathrm{RT}$$

6 $${T\Delta S}_{x}^{‡}={\Delta H}_{x}^{‡}-{\Delta G}_{x}^{‡}$$

Here, *R* is the ideal gas constant (8.314 J mol^–1^ K^–1^), *h* is the Planck constant (6.62 × 10^–34^ J s), *k*_B_ is the Boltzmann constant (1.381 × 10^–23^ J K^–1^).

#### 3.3.1 Energy Barriers for Association

For the association step between free αLa and AuNPs, Δ*G*_*a*_^‡^ values were distributed within a relatively narrow range (∼25.70–29.30 ± 0.01 kJ mol^–1^), indicating comparable energetic requirements for formation of the activated complex across surface chemistries. Additionally, a linear dependence of ln *k_a_* on 1/*T* was verified for the AuNPs@MPA and AuNPs@PEG systems, in agreement with the classical Arrhenius law, while the AuNPs@Cit system exhibited a polynomial fit, suggesting a mechanistic shift across the investigated temperature range. Elucidation of the molecular origins underlying these differences requires analysis of the enthalpic and entropic contributions associated with the transition state. In this context, Figure 5 presents the values of Δ*H*_*a*_^‡^ and *T*Δ*S*_*a*_^‡^ as a function of temperature for all evaluated complexes.

**Figure 5:**
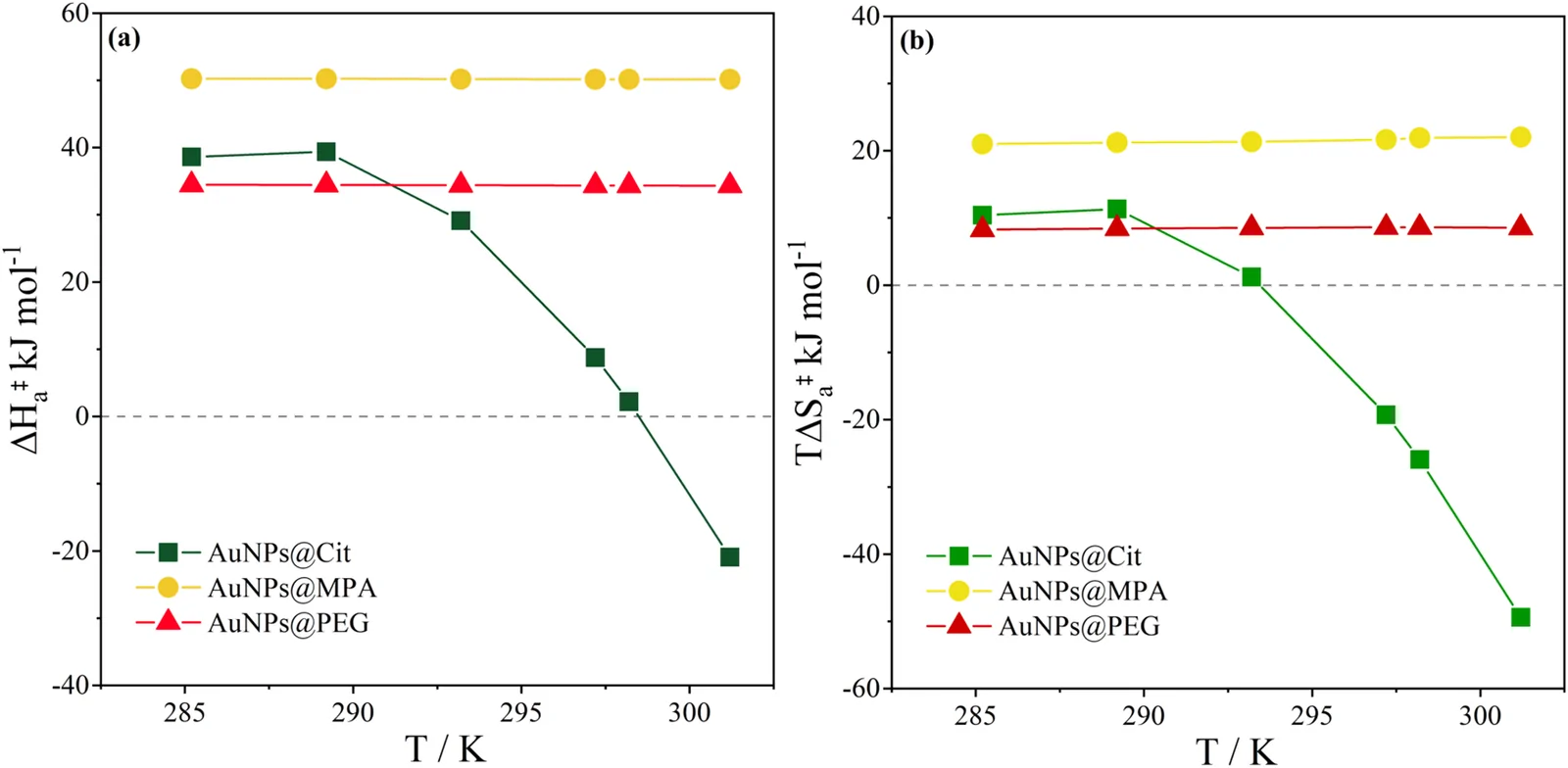
Temperature dependence of the activation enthalpy change (a), and activation entropy change (b) for the association process αLa interacting with surface-functionalized gold nanoparticles, within the 285.2–301.2 K range. Error bars representing the propagated uncertainties of the fitted parameters are smaller than the data markers and are not visible.

For AuNPs@Cit, Δ*H*_*a*_^‡^ and *T*Δ*S*_*a*_^‡^ exhibited a marked temperature dependence. This nonlinear behavior indicates that transition-state formation is not governed exclusively by diffusion and interfacial recognition, but probably involves additional structural reorganization steps, including partial conformational rearrangements of the adsorbed protein.^35^ In agreement, Li et al. demonstrated that the interaction of different proteins with AuNPs@Cit (∼10 nm) promotes alterations in secondary structure and may even compromise biological activity, as observed for lysozyme, trypsin, and pepsin.^36^

Accordingly, at lower temperatures, the positive values of Δ*H*_*a*_^‡^ and *T*Δ*S*_*a*_^‡^ suggest that the free-energy barrier may be influenced by unfavorable contributions arising from electrostatic repulsion between the interacting species, as well as by desolvation processes and counterion release during activated-state formation. As the temperature increases, the progressive reduction of these contributions may be associated with enhanced protein conformational flexibility. Such structural adjustments could facilitate the accommodation of αLa at the AuNP surface and favor the establishment of more effective intermolecular interactions (Figure 6a). Although these structural changes were not directly measured in the present study, hydrogen bonding and protein-metal electrostatic contacts represent plausible contributors to the observed inversion in the sign of Δ*H*_*a*_^‡^ and *T*Δ*S*_*a*_^‡^ at higher temperatures.

**Figure 6:**
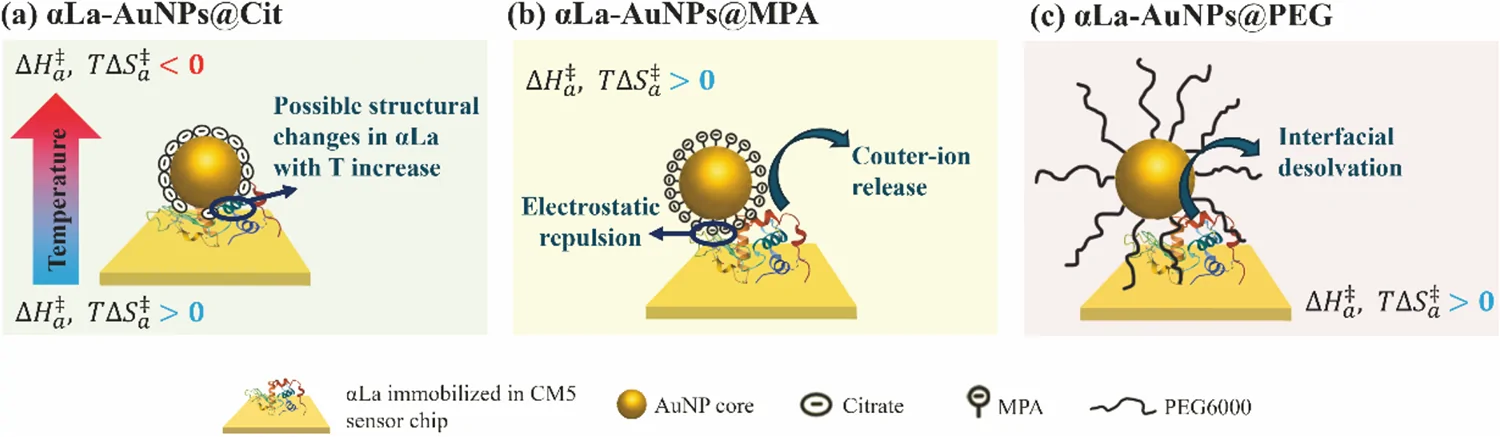
Proposed molecular representation of the association between immobilized αLa and AuNPs functionalized with (a) citrate, (b) MPA, and (c) PEG 6000, based on the experimentally determined activation parameters.

For AuNPs coated with MPA and PEG 6000, this nonlinear profile was not observed. Activation parameters remained confined to a narrow range across temperature, evidencing weak thermal dependence. The positive values of Δ*H*_*a*_^‡^ and *T*Δ*S*_*a*_^‡^ obtained over the entire temperature interval for both coatings indicate that the energetic barrier is predominantly governed by electrostatic repulsion and counterion release in the case of AuNPs@MPA (Figure 6b), and by interfacial desolvation contributions for AuNPs@PEG (Figure 6c). The fact that PEG 6000 carries no formal charge explains the lower values of Δ*H*_*a*_^‡^ and *T*Δ*S*_*a*_^‡^ relative to MPA over the whole temperature range and to citrate at *T* < 293 K, since the absence of electrostatic repulsion should reduce the energy required for protein–surface approach, leaving removal of structured water molecules from the polymeric layer as the main barrier contribution.

Unlike the citrate system, however, the presence of MPA or PEG on the AuNP surface does not appear to induce significant conformational perturbations in αLa, as increasing temperature does not translate into expressive variations in activation parameters. Citrate, due to its low molecular weight and predominant coordination via carboxylate groups, adsorbs onto the gold surface forming a structurally less organized interfacial layer, characterized by multiple binding modes and high interfacial mobility.^37,38^ This interfacial accessibility probably facilitates direct coupling between the protein and the metallic surface, since sulfhydryl (−SH) groups from cysteine residues display high chemical affinity for gold, establishing Au–S coordination interactions. For example, Wang et al. report studies showing that when bovine serum albumin (BSA) interacts with AuNPs, its disulfide bridges can anchor onto the nanoparticle surface via formation of multiple Au–S bonds. In contrast, lower-molecular-weight proteins lacking cysteine residues, such as ubiquitin, associate with citrate-coated AuNPs predominantly through short-range nonelectrostatic interactions, including hydrogen bonding.^39–41^

By comparison, MPA forms self-assembled monolayers chemisorbed through Au–S bonds, characterized by high surface packing density (∼6 molecules nm^–2^) and elevated structural stability,^42^ which shields the metallic surface and likely restricts direct protein adsorption. PEG, in turn, generates highly hydrated polymer layers that impose steric and entropic barriers to biomolecular approach.

#### 3.3.2 Energy Barriers for Dissociation

From eqs 3–6, the activation parameters for the dissociation process of the αLa-AuNPs complexes were calculated, and the values of the enthalpic and entropic contributions are shown in Figure 7 as a function of temperature.

**Figure 7:**
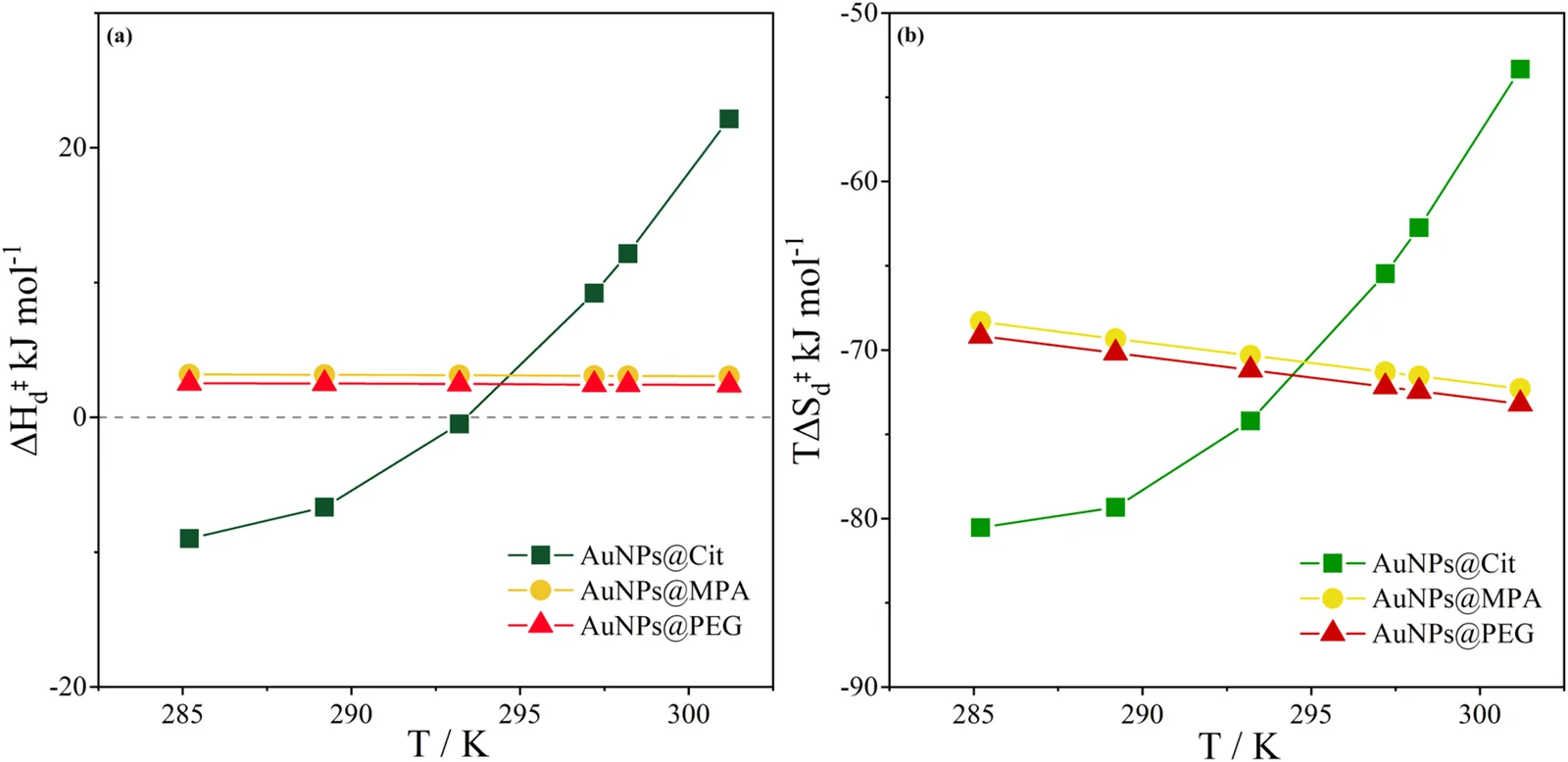
Temperature dependence of the activation enthalpy change (a), and activation entropy change (b) for the dissociation process αLa interacting with surface-functionalized gold nanoparticles, within the 285.2–301.2 K range. Error bars representing the propagated uncertainties of the fitted parameters are smaller than the data markers and are not visible.

For AuNPs@Cit, a pronounced nonlinear thermal dependence of the activation parameters is observed. The value of Δ*H*_*d*_^‡^ increases progressively with temperature, starting negative and turning positive at *T* > 293 K. Whereas *T*Δ*S*_*a*_^‡^ remains negative but becomes less unfavorable upon heating. A similar behavior was observed for the association step, indicating that dissociation of the AuNPs@Cit complex also requires conformational changes in αLa, resulting in an overall reduction in their degrees of freedom.

From the thermodynamic perspective, the relative magnitudes of Δ*H*_*d*_^‡^ and *T*Δ*S*_*d*_^‡^ provide insight into the structure of the stable complex. Low enthalpic barriers combined with strongly negative entropic terms indicate that the bound state is stabilized mainly by weak, distributed interactions and retains significant hydration and conformational freedom. In contrast, higher and positive Δ*H*_*d*_^‡^ values associated with less unfavorable entropy suggest a more tightly coupled interface, involving closer protein–surface contact and partial interfacial dehydration.

For the αLa-AuNPs@MPA complex, the activation parameters exhibit linear temperature dependence (Figure 7a), characterized by low Δ*H*_*d*_^‡^ values that remain slightly positive across the entire thermal range, and by strongly negative entropic terms that become progressively more unfavorable as *T* increases (Figure 7b). An analogous trend is observed for the αLa-AuNPs@PEG system. In both cases, this profile suggests that the dissociation step is governed predominantly by entropic contributions associated with interfacial reorganization. For MPA-coated AuNPs, the barrier may be associated with restructuring of the hydration network and the electrical double layer at the transition state, while in the PEGylated system, rearrangement of the solvation layer may play a more prominent role. These processes may impose a significant entropic penalty on complex dissociation without inducing substantial conformational alterations in the protein, which would be consistent with the behavior previously observed for the association step.

## 4 Conclusion

In this work, the kinetics and activation thermochemistry governing the interaction between α-lactalbumin and gold nanoparticles functionalized with distinct surface ligands (citrate, MPA, and PEG 6000) were systematically investigated using surface plasmon resonance to obtain association, dissociation, and activation parameters. The results demonstrate that αLa adsorption onto AuNPs occurs through a rapid and reversible kinetic regime, as evidenced by fast association and dissociation processes regardless of the surface coating employed. A clear dependence of kinetic behavior on ligand chemical identity was observed, with both association rate constants and residence times modulated by the interfacial physicochemical properties imposed by each functionalization. Activation analyses provided deeper mechanistic insight, suggesting that the energetic barriers governing complex formation arise from coupled contributions of interfacial desolvation and counterion release across all systems. Notably, citrate-coated nanoparticles exhibited additional temperature-dependent conformational reorganization of αLa, reflected in nonlinear activation parameter profiles. In contrast, MPA- and PEG-functionalized nanoparticles displayed a linear thermal dependence of activation parameters, consistent with adsorption mechanisms potentially dominated by electrostatic and hydration-mediated interactions, respectively. The thermodynamic signatures of dissociation further suggest that entropic contributions associated with interfacial and solvation-layer reorganization may be important determinants of complex stability. Collectively, these findings demonstrate that surface ligand engineering modulates adsorption kinetics, the energetic landscape of complex formation, and the molecular pathways underlying protein-nanoparticle interactions. This mechanistic understanding expands the physicochemical framework describing nanobiointerfacial phenomena and provides relevant insights for the rational design of functional nanomaterials in biosensing, nanomedicine, and biointerface engineering applications.

## Supplementary Material



## Notes

Statistical analysis and error propagation of the kinetic, residence-time, and activation parameters (Equations S1–S5); SEM micrograph of AuNPs showing their morphology and spatial distribution (Figure S1); SPR sensorgrams for the interaction between immobilized αLa and AuNPs@MPA (Figure S2), AuNPs@Cit (Figure S3), and AuNPs@PEG (Figure S4) at 285.2–301.2 K (PDF)

## ∥

Laboratório de Patologia de Sementes, Departamento de Fitopatologia, Universidade Federal de Lavras, Lavras, 37200–900, Brasil

## ⊥

Departamento de Química, Universidade Federal do Oeste do Pará, Santarém, 68040–070, Brasil
