# Supplementary material for: Surface Ligand-Dependent Kinetics of α‑Lactalbumin Interaction with Gold Nanoparticles Investigated by SPR
Source: ACS Omega. 2026 Sep 7;11(37):55082–92. doi: 10.1021/acsomega.6c01893 (PMC13613468; doi:10.1021/acsomega.6c01893)
Supplement: Supplementary file 1 [file ao6c01893_si_001.pdf]

Surface Ligand-Dependent Kinetics of α-Lactalbumin Interaction with Gold Nanoparticles Investigated by SPR

Carlos Antônio Ferraz Jr.[a], Isabela Araujo Marques[b†], Thalles Maranesi Pereira[a], Ygor Ribeiro Guimarães[b], Cínthia das Dores Aguiar[a], Hauster Maximiler Campos de Paula[b‡], Luciano Sindra Virtuoso[a], Nelson Henrique Teixeira Lemes[a], Ana Clarissa dos Santos Pires[c], Luis Henrique Mendes da Silva[b]*, Yara Luiza Coelho Zampier[a]*

[a]*Grupo de Pesquisa em Química de Coloides, Instituto de Química, Universidade Federal de Alfenas, Alfenas, 37130-000, Brasil.*

[b]*Advanced Thermokinetics of Molecular Systems (ATOMS) Group, Departamento de Química, Universidade Federal de Viçosa, Viçosa, 36570-000, Brasil.*

[c]*Laboratório de Termodinâmica Molecular Aplicada (THERMA), Departamento de Tecnologia de Alimentos, Universidade Federal de Viçosa, Viçosa, 36570-000, Brasil.*

*Email: yara.coelho@unifal-mg.edu.br; Phone: +55 (32) 9948-9915
*Email: luhen@ufv.br

[†]*Present Address: Laboratório de Patologia de Sementes, Departamento de Fitopatologia, Universidade Federal de Lavras, Lavras, 37200-900, Brasil.*

‡Present Address: Departamento de Química, Universidade Federal do Oeste do Pará, Santarém, 68040-070, Brasil.

Supporting Information

1. Statistical Analysis

The Standard Errors of the Fit (SE) for each kinetic parameter was derived directly from the standard error of the slope in the linear regression of k_{obs} versus [AuNPs], calculated using the parameter covariance matrix:

$$\mathrm{SE}_{\mathrm{k_a}} = \sqrt{\mathrm{Var}\left(\widehat{\mathrm{k_a}}\right)} = \sqrt{\sigma^2 (\mathrm{X^T X})_{11}{}^{-1}} \qquad \textbf{Equation S1}$$

where X is the design matrix containing the analyte concentrations ([AuNPs]) and

$$\sigma^2 = \frac{\sum\left(\mathrm{X_i} - \widehat{\mathrm{X}}_\mathrm{i}\right)^2}{\mathrm{N} - \mathrm{p}} \qquad \textbf{Equation S2}$$

Since the final value of k_d is reported as the average of the independent fits across all concentrations, the error ($\pm\ \mathrm{SE}_{\mathrm{k_d}}$) represents the propagated standard error of these individual regressions. It was calculated using Gauss's law of error propagation to combine the variances ($\pm\ \sigma^2_{\mathrm{k_{d,i}}}$) of each independent linear fit:

$$\mathrm{SE}_{\mathrm{k_d}} = \sqrt{\frac{\sum_{\mathrm{i=1}}^{\mathrm{N_{exp}}} \sigma^2_{\mathrm{k_{d,i}}}}{\mathrm{N^2_{exp}}}} \qquad \textbf{Equation S3}$$

where

$$\sigma^2_{k_{d,i}} = \mathrm{Var}(\widehat{k_d}, i) = \sigma^2 {(R_{diss}{}^T R_{diss})_{11}}^{-1}$$ **Equation S4**

$$\sigma^2 = \frac{\sum(\ln(R_{diss,i}) - \ln(\widehat{R}_{diss,i}))^2}{N - p}$$ **Equation S5**

Finally, the error bar for the complex residence time ($\tau_r = 1/k_d$) and the activation parameters were calculated using non-linear Gaussian error propagation.

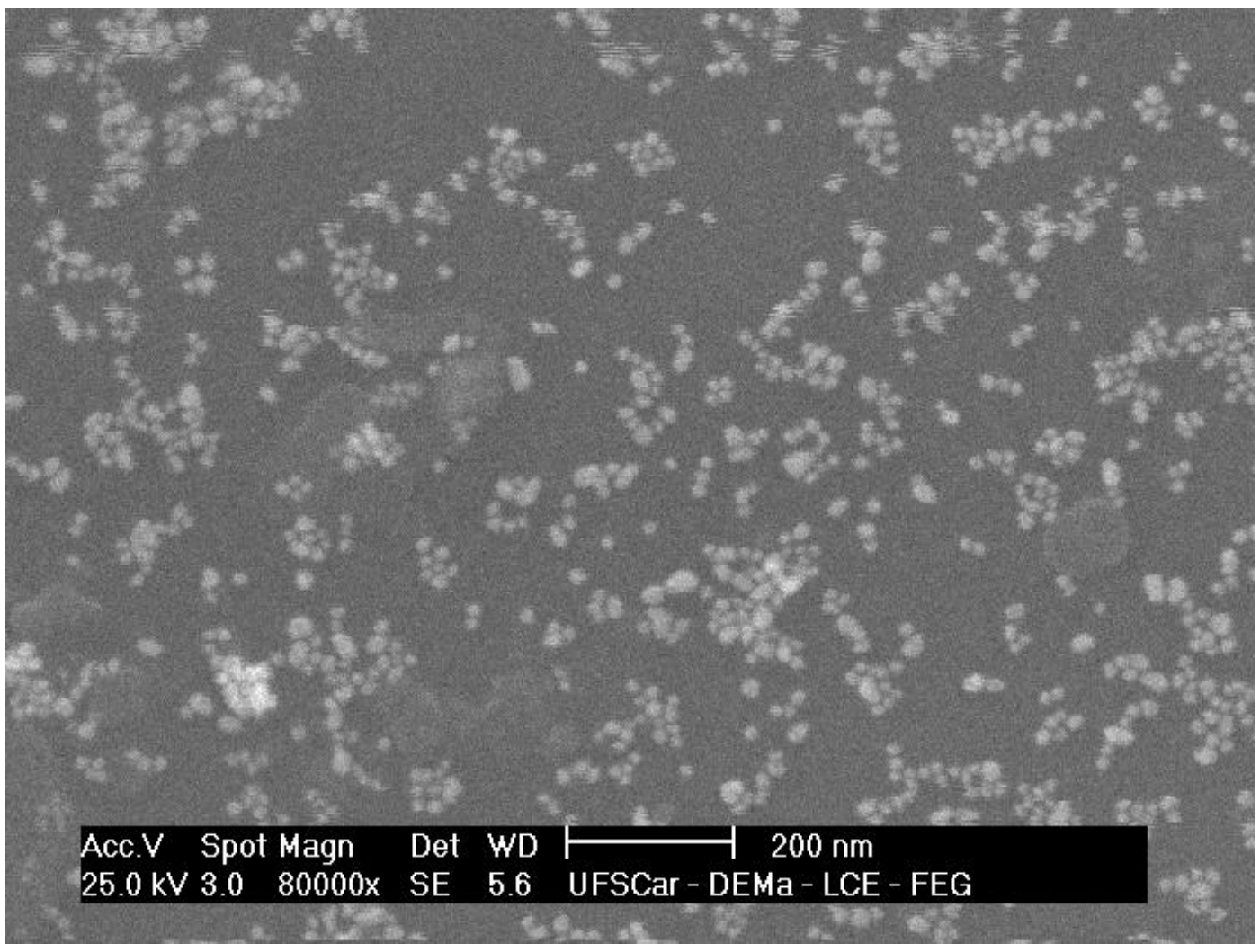

Figure S1. SEM micrograph of AuNPs showing their morphology and spatial distribution. Images were acquired in SE mode at 25 kV and 80,000× magnification. Scale bar: 200 nm.

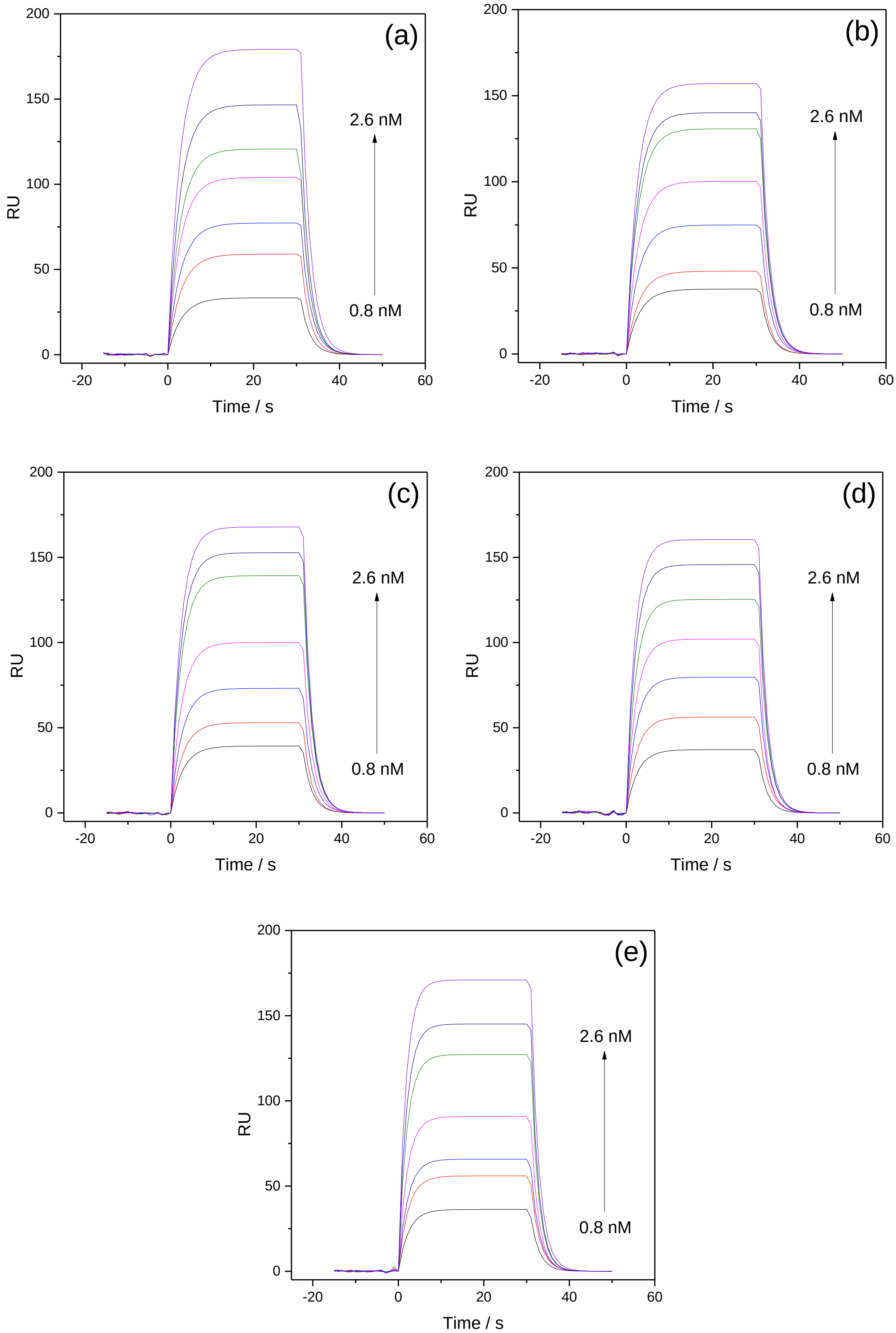

Figure S2. SPR sensorgrams for the interaction between immobilized αLa and AuNPs@MPA at (a) 285.2 K, (b) 289.2 K, (c) 293.2 K, (d) 297.2 K and, (e) 301.2 K.

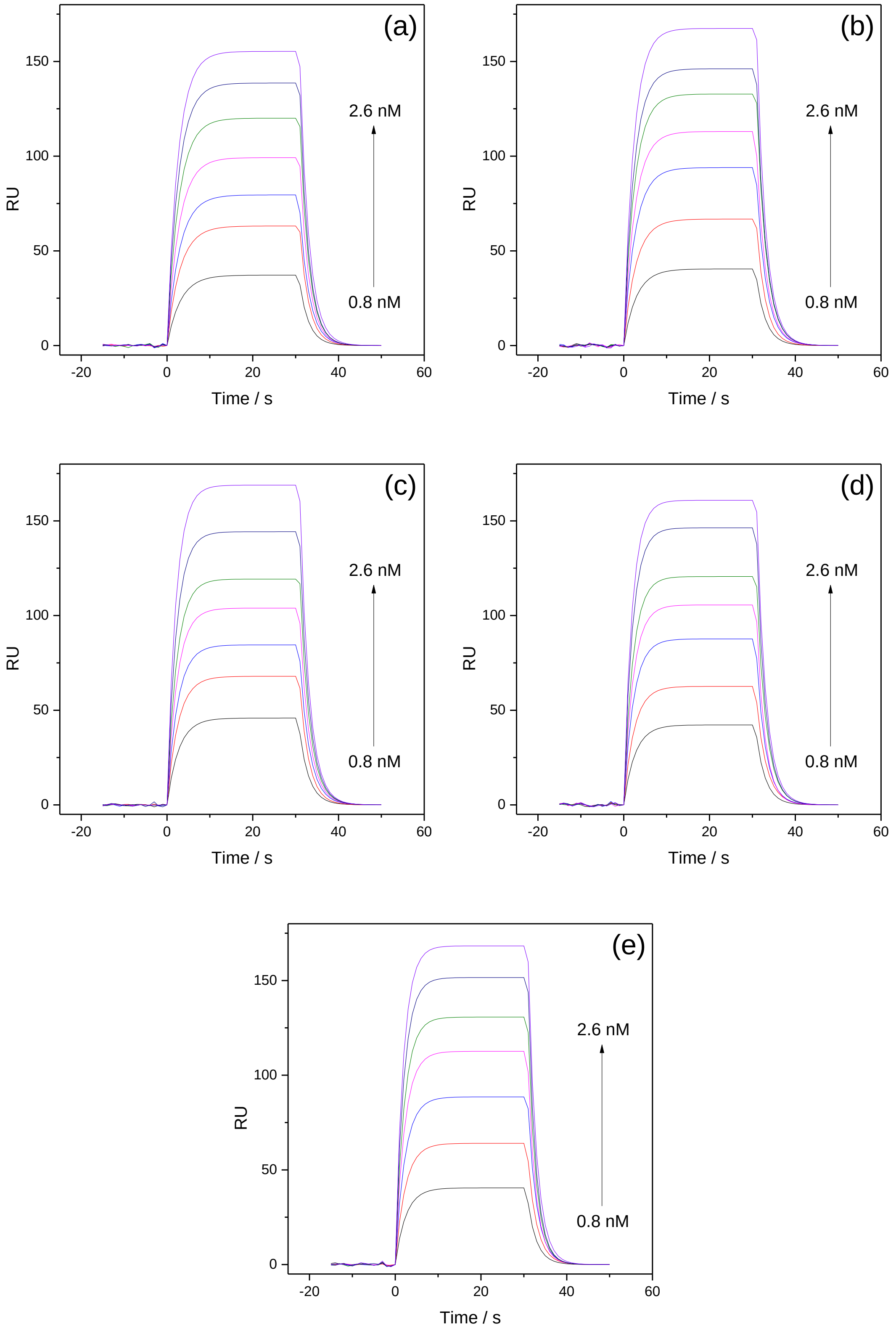

Figure S3. SPR sensorgrams for the interaction between immobilized αLa and AuNPs@Cit at (a) 285.2 K, (b) 289.2 K, (c) 293.2 K, (d) 297.2 K and, (e) 301.2 K.

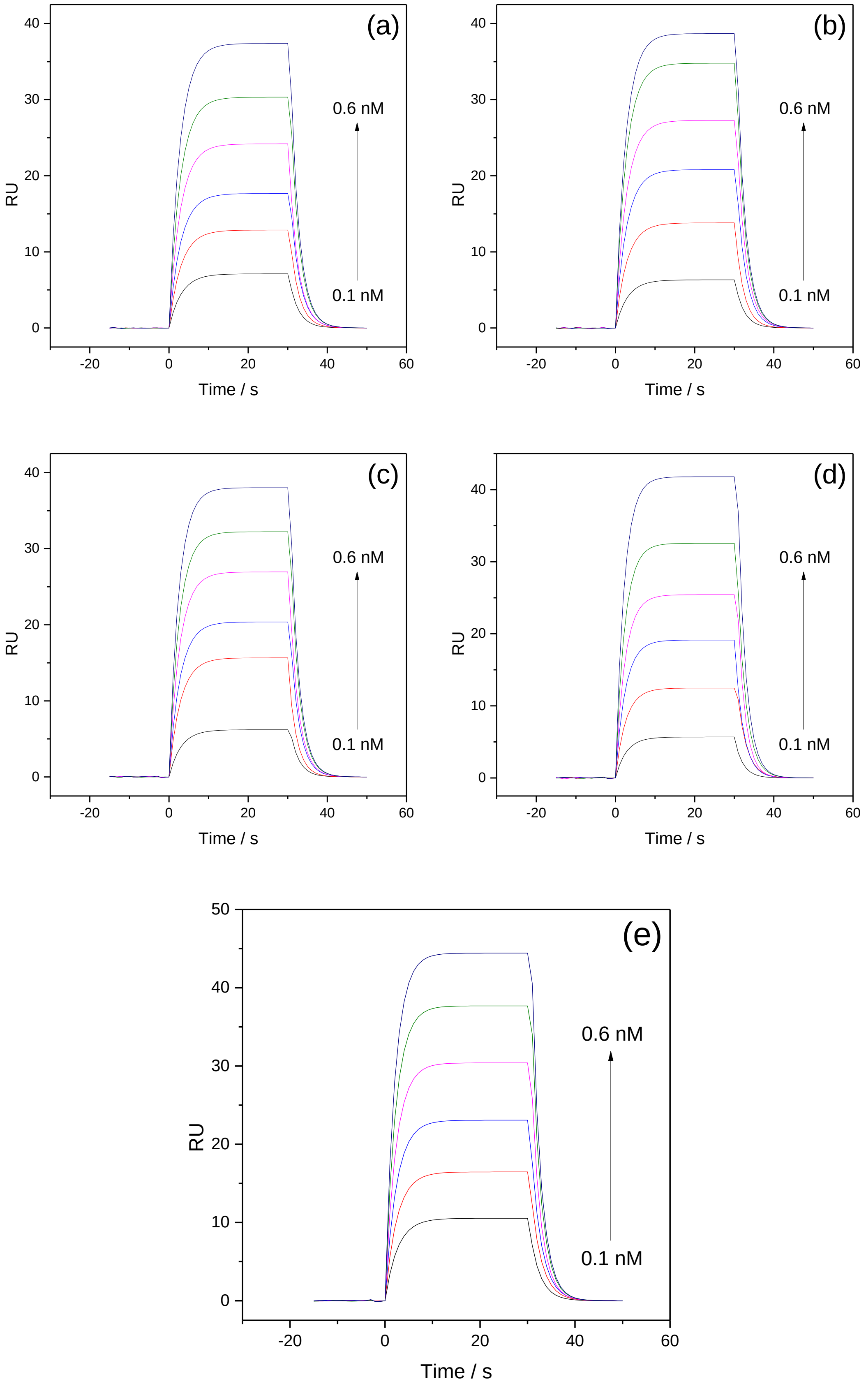

Figure S4. SPR sensorgrams for the interaction between immobilized αLa and AuNPs@PEG at (a) 285.2 K, (b) 289.2 K, (c) 293.2 K, (d) 297.2 K and, (e) 301.2 K.
